# Foreign Body in Nose—Failure in Diagnosis—Spontaneous Recovery

**Published:** 1871-09

**Authors:** C. T. Fenn

**Affiliations:** Chicago, Ill.


ARTICLE IV.—*Foreign Body in Nose—Failure in Diagnosis—Spontaneous Recovery.* By C. T. FENN, M.D., Chicago, Ill.

November 25, 1870. Anna B., aged two years, had been strangely affected during the previous fortnight. She grew irritable by day and restless by night, often awaking and crying; her breathing was difficult and appeared to be obstructed. She rubbed her nose frequently. Her symptoms gradually got worse. Coryza became marked. The nasal secretion was scanty and sometimes fetid. Her condition during the third week excited alarm. Large vesicular sores broke out on her face. Extensive aphthous patches formed in her mouth. The lips swelled; the salivary glands became unusually active. The nose was enlarged; the nostrils were contracted. A scanty, purulent and excessively fetid discharge came from them.

The treatment was nutrient and detergent, but unavailing. The worst symptoms of coryza continued for many weeks, and were finally tolerated, such very slight alleviation was wrought by time.

The parents were inconsolable. This youngest and fondest of several beautiful children had become an object of pitiable suffering. In their endeavor to find a cause it was presumed to have been vaccination once performed.

The mother had had disease of the shoulder-joint and had undergone resection of the head of the humerus therefor. Several years had elapsed since the operation, and her recovery had been perfect, except as to motion, but the fact was construed to indicate a diathesis. The child was believed to be *scrofulous.*

June 10, 1871. At last all doubts were solved by an occurrence as unexpected as it was satisfactory. The child, while picking her nose, withdrew a plug of woolen cloth. When washed and smoothed, the stuff proved to be green and blue plaid, of which children's dresses are made, and measured three inches long by three-fourths of an inch wide. The green was faded. A week later she relieved herself, by the same method, of another plug, of black silk, equally large, from the opposite side. The cure followed immediately, seven months after the appearance of the disease.

